# Towards whole life-stage naked clam aquaculture: integrating settlement, growth, and nutrition of *Lyrodus pedicellatus*

**DOI:** 10.1007/s10499-026-02525-y

**Published:** 2026-04-01

**Authors:** J. Reuben Shipway, Max Lancaster, Tatiana Zúñiga-Burgos, Konstantinos P. Papadopoulos, Payam Mehrshahi, Alison G. Smith, David F. Willer

**Affiliations:** 1 Naked Clam Limited, 24 Albert St, Cambridge, CB4 3BE England; 2 The International Marine Wood-Borer Network (IMWBN), Birmingham, UK; 3 Faculty of Science & Engineering, Plymouth University, Drake Circus, Plymouth, PL4 8AA England; 4 Department of Plant Sciences, University of Cambridge, Downing St, Cambridge, CB2 3EA England; 5 GreenCoLab—Associação Oceano Verde, University of Algarve, Campus de Gambelas, Faro, 8005-139 Portugal; 6 Department of Zoology, University of Cambridge, Downing Street, Cambridge, CB2 3EJ England

**Keywords:** Food production, Sustainability, Planetary boundaries, Net zero, Blue food, Teredinidae

## Abstract

Rapidly growing global demand for nutrient-dense foods is currently testing planetary boundaries, while global food systems are failing to alleviate a persistent burden of malnutrition in human populations. Naked clams (Teredinidae) have recently emerged as a promising candidate to address these deficiencies, offering high-nutrient, low-environmental impact “blue food” production. While previous research demonstrated the viability of maintaining adult naked clams in modular systems, full life-cycle culture and quantified growth performance remained unexplored. This study evaluates the capacity of a modular, non-flow-through static-tank system to support the full development of the naked clam species *Lyrodus pedicellatus* from larval settlement to maturity. We quantified growth responses to a factorial combination of different wooden habitat structures (sheet vs. square dowel panels) and microencapsulated feed regimes (Biobullets). The results demonstrate that continuous dietary supplementation significantly enhances growth, with “full feed” individuals exhibiting significantly greater length and wet weight compared to no-feed controls, regardless of wood structure. Nutritional profiling revealed that *L. pedicellatus* possesses high concentrations of vitamin B12 (~ 70 µg g⁻^1^ dry weight) and beneficial fatty acids, including the neuroprotective nervonic acid (C24:1n9). Although absolute growth rates were lower than those observed in wild contexts, the tractability of *L. pedicellatus*—characterised by continuous breeding and rapid generation times—establishes it as an ideal model organism for aquaculture optimisation. These findings suggest that naked clam aquaculture may be feasible at commercial scale, opening a pathway for transforming lignocellulosic waste into functional, nutrient-dense protein within a circular bioeconomy.

## Introduction

Rapidly growing global demand for nutrient-dense foods is colliding with planetary boundaries and a persistent burden of malnutrition. At least two-thirds of the world’s population are estimated to have inadequate intakes of essential vitamins and minerals, while at the same time, the global food system accounts for roughly one-third of total anthropogenic greenhouse gas emissions, driven by agriculture, land-use change, and associated supply chains (Crippa et al. 2021; Passarelli et al. 2024; Ranganathan et al. 2018; WWF, 2024). Aquaculture, and blue food more broadly, have been highlighted as critical to improving food security and nutrition while reducing environmental impacts (ASC, 2023; Wang and Liu 2022; Willer et al. 2024). Yet, dominant finfish and crustacean systems can be highly resource-intensive, relying on wild fish inputs, soy, and other crop-derived feeds, and often generate nutrient pollution (Rust et al. 2011; Froehlich et al. 2018; Jones 2021). Diversifying blue food portfolios with low-input, high-nutrient species is therefore a central priority for humanity’s dietary needs and environmental sustainability (Willer et al. 2021; Campanati et al. 2022).

Bivalve aquaculture already offers a compelling low-feed, low-carbon option (Froehlich et al. 2018; Willer et al. 2021), but conventional mussel, clam, and oyster systems remain constrained by biological and economic limits. A substantial fraction of assimilated energy is invested in shell rather than edible tissue, and production is often limited to coastal sites with suitable water quality and infrastructure (Kaschner et al. 2016; Gawel et al. 2023; Willer et al. 2020, 2021). Naked clams (Teredinidae)—historically known as shipworms—have recently emerged as a radically different bivalve model for food production (Willer and Aldridge 2020; Willer et al. 2023; Poon et al. 2025). The term “Naked Clam™” is a registered trademark referring to teredinid bivalves (commonly known as shipworms) used in aquaculture and food production contexts; throughout this manuscript we use the term “naked clams” as a descriptive shorthand for members of the family Teredinidae. These wood-boring molluscs possess highly reduced shells and elongated bodies, diverting energy away from shell formation and into exceptionally rapid soft tissue growth (Willer et al. 2023; Shipway et al. 2024; Poon et al. 2025). Meta-analyses suggest that many teredinid species grow several times faster than farmed blue mussels (*Mytilus edulis*) and can reach much larger final sizes, including species exceeding 1 m in length (Poon et al. 2025). Symbiotic bacteria in their gills and digestive system enable lignocellulose digestion and nitrogen fixation, allowing naked clams to transform low-nutrient-value wood into nutrient-dense protein (Altamia et al. 2020; Pesante et al. 2021; Stravoravdis et al. 2021; Goodell et al. 2024).

Recent work has begun to establish naked clams as a novel, sustainable seafood. Willer et al. (2023) developed the first modular static-tank system for maintaining *Teredo navalis* on wooden substrates supplemented with microencapsulated algal feeds (Biobullets). They demonstrated that adult naked clams assimilate these feeds, which can be used to fortify tissue concentrations of omega-3 fatty acids (EPA, DHA) and vitamin B12 beyond those found in blue mussels (*Mytilus edulis*) (Willer et al. 2023; NOAA Fisheries 2022). This, together with evidence of high protein and favourable fatty acid profiles (Willer et al. 2023; Poon et al. 2025) and analyses of self-reported consumer experiences on social media indicating that many individuals who have tried naked clams report enjoying them (Shipway et al. 2024), positions the group as a promising candidate for nutrient-rich blue food production. In parallel, detailed laboratory protocols have demonstrated that some species can be maintained, propagated, and induced to spawn in closed aquarium systems for biological research and symbiosis studies (Shipway et al. 2020; Flatau et al. 2025), but these systems were not designed to optimise growth, feeding regimes, or production performance for aquaculture.

Current naked clam aquaculture remains largely at a conceptual and pilot stage. In the initial *T. navalis* study, clams were pre-grown in the wild and only maintained in the modular system (Willer et al. 2023). Growth rates were not quantified, life-stage rearing (from larval settlement to maturity) was not demonstrated, and only a single species was nutritionally profiled. It therefore remains unclear whether naked clam growth can be reliably driven in simple, non-flow-through systems, how growth responds to microencapsulated feed regimes, and whether high nutritional value is general across the family rather than a peculiarity of *T. navalis*. The recent global review by Poon et al. (2025) highlighted these gaps explicitly, collating growth and size data for 29 naked clam species and showing that many exhibit extremely rapid growth and large size potential compared with mussels, yet also noting the near-absence of species-specific nutritional profiling and experimental aquaculture trials. That review identified the *L. pedicellatus* morphospecies complex as containing potentially important aquaculture candidates based on life-history traits and broad historical records, although recent phylogenetic work indicates that *L. pedicellatus* sensu lato comprises multiple cryptic lineages rather than a single cosmopolitan species (Borges et al. 2012; Borges and Merckelbach 2018; Treneman et al. 2026).

Accordingly, historical physiological and reproductive studies attributed to *L. pedicellatus* may refer to the broader morphospecies, *L. pedicellatus* sensu lato, rather than *L. pedicellatus* sensu stricto (Borges and Merckelbach 2018; Treneman et al 2026). Nevertheless, material identified as *L. pedicellatus* has been the subject of several detailed physiological investigations, making it one of the few teredinid species for which detailed physiological studies exist (Turner and Johnson 1971; Gallager et al. 1981; Pesante et al. 2021). These studies also describe a reproductive mode particularly well suited for laboratory culture. Material historically identified as L. pedicellatus has been reported to breed continuously at water temperatures between 14 and 24 °C (Eckelbarger and Reish 1972; Carlton 1979), whereby fertilised embryos are brooded on the gill, and larvae are subsequently released at an advanced stage into the water column, where they remain planktonic for only 2–24 h before settlement and metamorphosis (Turner and Johnson 1971; Eckelbarger and Reish 1972). This short planktonic and quick settlement stage at amenable temperatures makes *L. pedicellatus* ideal for controlled settlement onto experimental substrates. Despite possessing traits that are well aligned with aquaculture production, *L. pedicellatus* has not yet been evaluated in an aquaculture context, and no nutritional profile has been reported for this species.

Here we address three critical gaps. First, we test whether the modular static-tank system originally developed for *T. navalis* (Willer et al. 2023) can support the full life cycle of *L. pedicellatus*, from larval settlement (from both induction and natural spawning) through to sizes consistent with sexual maturity (Flatau et al. 2025), without flow-through. Second, we quantify growth responses (length and wet weight) to a factorial combination of microencapsulated feed regimes and different wood habitat structures, providing the first experimental evidence on how Biobullets and wood type affect growth in a primarily xylophagous bivalve. Third, we provide the first full nutritional profile for *L. pedicellatus* (macronutrients, vitamin B12, and fatty acid methyl esters, FAMEs) and compare it qualitatively with published data for *T. navalis* and blue mussels *Mytilus edulis* (Lander et al. 2012; Willer et al. 2023).

## Materials and methods

### Species choice and collection

*Lyrodus pedicellatus* specimens were originally collected from solid wooden panels (softwood pine), measuring 20 × 15 × 2.5 cm, deployed off the Coxside Pontoon (50.365842, − 4.131959), University of Plymouth Marine Station, Plymouth, UK. Specimens were morphologically identified as *L. pedicellatus* using the taxonomic keys from Turner (1971). We acknowledge that morphology alone cannot exclude other members of the *L. pedicellatus* complex, but recent phylogenetic work places UK material within the north-east Atlantic *L. pedicellatus* clade, supporting the use of the present material as a working model for *L. pedicellatus* sensu stricto. Larvae produced by adults from these colonised seed panels were used to populate the subsequent experimental set-up outlined in the “Cultivation and wooden growth structure” section below.

### Diet

Four dietary regimes were applied to *L. pedicellatus* over the course of the growth experiment. These comprised the following: (1) no feed across the experiment; (2) supplementation beginning 1 week before the end of the experiment; (3) supplementation beginning 1 month before the end of the experiment; and (4) supplementation throughout the experiment. For clarity, these are hereafter referred to as “No Feed”, “One Week”, “One Month,” and “Full Feed”, respectively. The feed was a 20:40:40 blend of three Biobullet formulations designed for naked clam integration and consumption (*Schizochytrium*, *Nannochloropsis* and *Tetraselmis* Biobullets, TasteTech, UK), as in Willer et al. (2023).

Bivalves exposed to high particulate concentrations can expel indigestible particles as pseudofaeces, which may obstruct siphons and impair growth and feed assimilation (Wisely and Reid 1978; Palmer and Rutherford 2011; Willer and Aldridge 2017). Because naked clams draw both water and feed through their siphons, feed dose had to be chosen to minimise pseudofaeces production while still providing sufficient nutrition. The ideal ration for many bivalves is approximately 3% of body weight per day (Helm and Bourne 2004), and previous work on oysters suggests that particle concentrations around 2 mg L⁻^1^ facilitate minimal pseudofaeces formation (Wisely and Reid 1978; Palmer and Rutherford 2011). A conservative feed concentration of 5 mg L⁻^1^ day⁻^1^ was selected as an informed estimate intended to minimise pseudofaeces production while ensuring sufficient particle availability for all clams within the tanks, as body weights of *L. pedicellatus* across life stages are not currently documented.

The Biobullets used in this study had an average particle diameter of 46 µm, with 90% of particles in the size range of 10–100 µm.

### Cultivation and wooden growth structure

**Fig. 1:**
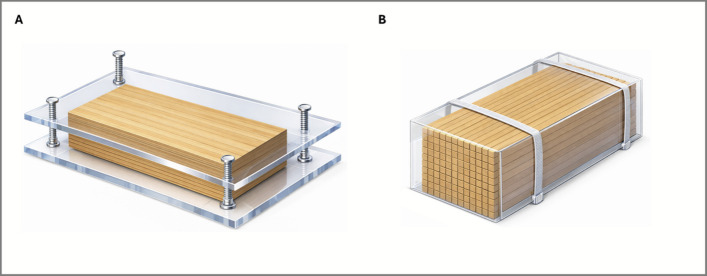
Experimental wooden growth structures used for *Lyrodus pedicellatus* cultivation. **A** “Sheet” panel modified from Manyak (1982). **B** Square dowel panel developed in this study. Both structures were designed to facilitate larval settlement, burrowing and subsequent extraction. In panel **B**, the PVC sheath is illustrated as transparent to reveal the internal arrangement of the square dowels; in practice, standard opaque PVC guttering was used. The sheath is secured with adjustable cable ties and intentionally left unsealed along one side to allow removal of the wooden assembly and to accommodate minor expansion of the wood when saturated; this is shown schematically as a small gap along the left side of the panel

Laboratory experiments were conducted between 07/02/2024 and 13/07/2024 in a 20 °C temperature-controlled room at the University of Plymouth, UK. Breeding procedures ran from 07/02/2024 to 08/04/2024. Existing stock of *L. pedicellatus* within seed panels was distributed among four 20-L tanks, each filled with 15 L of filtered seawater and aerated with a large airstone. Salinity was monitored using a handheld refractometer and maintained within the normal seawater range (~ 35 PSU). Evaporation was minimised by placing lids on the tanks, and any water loss between water changes was compensated by topping up with deionised water to maintain stable salinity. Two wood structures, hereby referred to as “square dowel” and “sheet” panels, were designed to perpetuate the naked clam culture (Fig. 1). Sheet panels (Fig. 1A) were developed from a modified design after Manyak (1982), whereby thin sheets of wood, each 1 mm in thickness, were stacked to form a solid wooden panel measuring 20 × 15 × 2.5 cm (length × width × thickness). This wooden core was sandwiched between two clear plastic barrier plates made from transparent Perspex, each measuring 22 × 17 cm (extending 1 cm beyond the wooden core in both length and width) and 5 mm thick. The assembly was secured using stainless steel screws that passed through the plastic cover only and were fastened at the underside with bolts, without entering the wooden core. Square dowel panels (Fig. 1B) were constructed from square-section wooden sticks, each measuring 15 cm in length and 0.5 × 0.5 cm in cross-section, which were tightly packed together to form a solid wooden block measuring 15 × 8 × 8 cm (length × width × thickness). The sticks tessellated fully, creating a contiguous wooden core with no gaps between individual elements. The assembled wooden core was enclosed within a removable plastic barrier sheath constructed from commercially available square PVC guttering, cut to size so that it enclosed all faces of the assembly except for one exposed surface, corresponding to the terminal ends of the wooden sticks (0.5 × 0.5 cm). These exposed end-grain surfaces provided the settlement interface for naked clam larvae. The sheath was intentionally left unsealed along one side to allow for easy removal and inspection, and to accommodate minor expansion of the wood when saturated. The assembly was secured using adjustable plastic cable ties, which tightened the sheath around the wooden core keeping the square dowels in place. For both the square dowels and sheets, the wood grain ran longitudinally along the length of the structure, such that the exposed surfaces were predominantly longitudinal grain, with end grain exposed only at the cut ends.

Twenty units of each wood type were evenly distributed across the breeding tanks. No feed was provided during the breeding period to avoid confounding juvenile growth. Half-water changes were performed weekly, and wood panels were gently cleaned in situ within the tanks using a soft-bristled brush underwater to remove biofilm and faecal pellets without disturbing settled larvae.

When breeding did not occur naturally, heat shock was used to stimulate larval release, following established methods for inducing spawning and gamete release in bivalves (Helm and Bourne 2004). Breeding blocks were placed in a water bath, and the temperature was raised from 20 to 30 °C over 10 min, maintained at 30 °C for 35 min, and then reduced back to 20 °C over a further 10 min. This treatment successfully stimulated larval release, after which water containing free-swimming larvae was redistributed among the breeding tanks.

### Growth

Growth experiments took place between 08/04/2024 and 12/07/2024 in the same 20 °C control room. After the breeding period, wood blocks were removed from breeding tanks and transferred into a modular aquaculture system based on Willer et al. (2023). Twenty static-tank set-ups were established, each containing one unit of each of the two wood types. Tanks were filled with 2 L of artificial seawater and aerated with a large airstone. Tanks were randomly assigned to one of the four feeding regimes, yielding five replicate tanks per diet.

Feeding occurred as twice-daily half-feeds at 09:00 and 15:00 every Monday to Friday throughout the experiment. This schedule was used to avoid oversaturation and pseudofaeces production and to maintain a more constant particle concentration analogous to natural environments (Palmer and Rutherford 2011; Ye et al. 2022). Each half-feed was first dispersed by stirring the Biobullets into 200 ml of seawater removed from the tank, which was then returned to improve particle distribution.

Blocks that produced no faeces—indicating a lack of larval settlement—were transferred to separate observation tanks for 2 weeks to minimise skew in growth data from late settlement. If faecal pellets subsequently appeared, wood was returned to its original tank. If no faecal pellets were produced, indicating no settlement, the wood was removed from the experiment. Half-water changes were conducted weekly, and wood blocks were routinely cleaned to remove excess faecal pellets, uneaten feed, and accumulated biofilm where present.

### Naked clam extraction

Naked clams were extracted from the square dowel and sheet panels as follows. For square dowel panels, cable ties were cut to loosen the plastic barrier allowing removal of individual square dowels. Naked clams were then carefully extracted from individual square dowels using a small hammer and chisel. For sheet panels, the plastic barrier plate was first removed by loosening the screws, and animals were then extracted by peeling off individual sheets of wood to reveal naked clam burrows. Individuals could then be carefully removed using a fine paintbrush. Extractions took place between 12/07/2024 and 13/07/2024. After extraction, any external calcium carbonate tube was removed. Wet weight (mg) was measured on blotted (removing excess seawater) individuals using an analytical balance, and burrow length (mm) was measured using digital callipers, with every single individual mesured. To evaluate the practicality of each habitat type, the time required to dismantle each wood unit and extract all clams was recorded. Total clam counts by wood structure and feed combination were 28 for full feed dowell, 75 for Full Feed sheet, 4 for No Feed dowell, 53 for No Feed sheet, 0 for One Week dowell, 21 for One Week sheet, 3 for One Month dowell, and 51 for One Month sheet.

### Nutritional analyses

Following length and weight measurements, all clams within each wood structure-feed type combination were pooled to form three biological replicates of at least 100 mg fresh weight per wood structure-feed type combination, with a minimum of three individuals per replicate. Due to limited biomass, a third biological replicate could not be obtained for both the One Week and No Feed treatments, so there were only 2 biological replicates in these cases. Replicates were freeze-dried (benchtop Edwards Modulyo 4 K, − 50 °C condenser) for 48 h and homogenised in a bead-mill tissue lyser (Qiagen TissueLyser II, 2 min at 25 Hz), prior to analysis.

#### Biochemical composition

Biochemical composition was determined using Fourier transform infrared (FT-IR) spectroscopy following Willer et al. (2023), using a Spectrum Two, PerkinElmer, Germany. Approximately 3–5 mg of finely powdered freeze-dried tissue was pressed on the crystal surface (iATR reflectance cell with a DTGS detector), and scans (wavenumber range of 4000–450 cm^−1^ at a resolution of 4 cm^−1^) were recorded and baseline-corrected using Spectrum (version 10, PerkinElmer, Germany). Approximately 3 mg of dried biomass per sample were analysed, and three spectra (technical replicates) were obtained per biological replicate. Baseline-corrected peak areas were used to calculate FT-IR peak area ratios (carbohydrate:protein and lipid:protein), which were converted to component ratios as a percentage of dry weight using existing calibration curves (Willer et al. 2023). Protein, carbohydrate, and lipid content were expressed as % dry weight after determining absolute protein content via the Pierce™ Modified Lowry Protein Assay. Approximately 2.5 mg of dried clam tissue were used per assay, with three technical replicates per sample. Colour development was read at 750 nm.

#### Vitamin B12 analysis

Vitamin B12 (cobalamin) content was quantified using a bioassay method developed by Papadopoulos et al. (2023). For each biological replicate, three extractions (technical replicates) were prepared by resuspending 5 mg of dried biomass in 5-ml sterile deionised water, then splitting into three × 1.5 ml aliquots. Vitamin B12 was extracted by boiling for 20 min and cooling. Each extract was diluted tenfold in sterile water in duplicate on a 24-well plate and inoculated with the B12-dependent *Chlamydomonas reinhardtii* strain metE7 (Bunbury et al. 2022). B12 content was calculated relative to cobalamin standards using a polynomial calibration model (Papadopoulos et al. 2023).

#### Lipid extraction and FAME analysis

Lipids were extracted from approximately 3 mg of dried biomass using a 2:1 chloroform:methanol solution, with two or three technical replicates per sample depending on biomass availability. Samples were sonicated on ice (4 °C) for 30 min. Phase separation was induced by adding deionised water, vortexing, and centrifugation at 4 °C for 3 min. The chloroform phase was collected, evaporated, and resuspended in 200 µl n-heptane, and transferred to glass GC vials.

FAMEs were prepared by transesterification of 50 µl aliquots with 3 ml of 2.5% H₂SO₄ in methanol, followed by vortexing and incubation at 60 °C for 4 h. After cooling, reactions were quenched with 3 ml 1:1 deionised water and hexane, and phases were separated by centrifugation at 2000 g for 3 min. The hexane phase was removed and evaporated, and the FAME extracts were resuspended in 120 µl n-heptane, transferred to GC/HPLC vials, and stored at − 70 °C prior to GC analysis.

FAME profiles were obtained by GC–MS following the method in Willer et al. (2023). Injection volume was 1 µl for most samples and 2 µl for No Feed samples to strengthen peak signals. FAMEs were identified by co-elution with a 37-component FAME standard mix (Supelco), with peaks assigned based on retention times. Relative abundances were expressed as the percentage of total identified FAMEs.

### Statistical analysis

Statistical analyses of growth and extraction time were conducted in RStudio (RStudio Team 2020) using the packages car (Fox and Weisberg 2019), FSA (Ogle et al. 2023), rcompanion (Mangiafico 2024), ggplot2 (Wickham 2016), and ggpubr (Kassambara 2023). A two-way ANOVA was initially considered for analysing length and weight but assumptions of homogeneity of variance could not be met, even after transformation, as indicated by Levene’s tests. A non-parametric Scheirer-Ray-Hare test was therefore used to assess the effects of feeding regime, wood structure, and their interaction on length and weight. Post hoc pairwise comparisons were performed using Dunn tests. To compare extraction times between sheet and square dowel structures, a one-way ANOVA was applied after confirming homogeneity of variance with Levene’s test. Nutritional data (protein, carbohydrate, lipid, vitamin B12, and fatty acid classes) were compared among feeding regimes using one-way ANOVA with Tukey–Kramer post hoc tests, conducted in Microsoft Excel 2021. All nutritional datasets met homogeneity of variance assumptions based on Levene’s tests.

## Results

### System performance and life-cycle completion

Larvae of *L. pedicellatus* successfully settled from the plankton onto sheet and square dowel wood structures in the modular static-tank system and developed into burrowing juveniles and larger adults over the 96-day experiment. Individuals exhibited sustained post-settlement growth within a closed, non-flow-through system, demonstrating that the modular design supported development beyond early juvenile stages. By the end of the experiment, individuals had reached sizes consistent with the onset of adulthood, as defined by prior culture studies, although direct evidence of reproductive activity or brood pouch development was not assessed in this study. These results demonstrate that a simple static modular system can support settlement and prolonged growth of *L. pedicellatus* without continuous flow-through, extending existing laboratory culture approaches by integrating settlement, growth, and feeding experiments within a single closed system.

### Growth responses to feed and wood structure

**Fig. 2:**
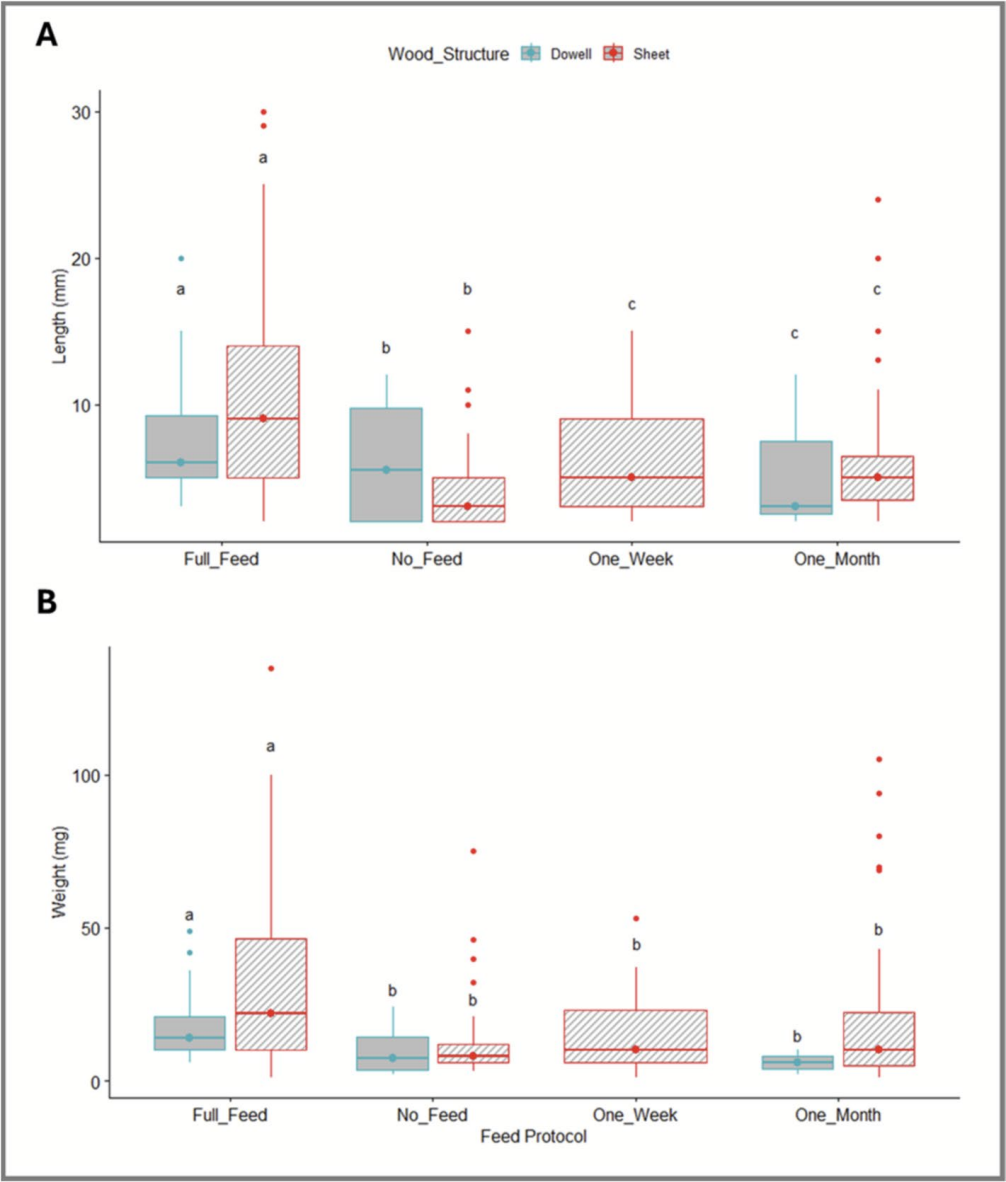
Growth responses of *Lyrodus pedicellatus *under different habitat structures and Biobullet feeding regimes. Length (**A**) and wet weight (**B**) of *L. pedicellatus* across experimental treatments, shown as boxplots. Boxes represent the 25th and 75th percentiles, with the central line indicating the median. Letters denote post hoc groupings, where shared letters indicate no significant difference between groups (*p* > 0.05). Total numbers recovered per wood structure × feed combination were as follows: Full Feed dowel *n* = 28, Full Feed sheet *n* = 75, No Feed dowel *n* = 4, No Feed sheet *n* = 53, One Week dowel *n* = 0, One Week sheet *n* = 21, One Month dowel *n* = 3, and One Month sheet *n* = 51

Median naked clam length under the Full Feed regime was significantly greater than under all other feeding regimes (Fig. 2A). In contrast, individuals in the No Feed regime were significantly shorter than those in all other treatments, while clams reared under One Week and One Month feeding regimes exhibited intermediate lengths that did not differ significantly from each other. Statistical analysis confirmed that feeding regime had a significant effect on median length (Scheirer-Ray-Hare test, H(3) = 41.175, *p* < 0.05), whereas wood structure had no effect (H(1) = 0.67, *p* = 0.41), and there was no feed × structure interaction (H(2) = 1.341, *p* = 0.51). Dunn post hoc tests showed that Full Feed clams were significantly longer than all other treatments (*p* < 0.05), No Feed clams were significantly shorter than all others (*p* < 0.05), and One Week and One Month treatments did not differ (*p* = 0.847). Overall, increased feed intensity resulted in larger individuals irrespective of wood structure.

Patterns in wet weight mirrored those observed for length. Median wet weight under the Full Feed regime was significantly greater than under all other feeding regimes (Fig. 2B), while clams in the No Feed treatment were significantly lighter than all others. Individuals in the One Week and One Month treatments again showed intermediate weights that did not differ significantly from each other. Feeding regime had a significant effect on median wet weight (H(3) = 34.448, *p* < 0.05), whereas wood structure did not (H(1) = 2.210, *p* = 0.13), and no feed × structure interaction was detected (H(2) = 0.907, *p* = 0.63). Dunn post hoc tests confirmed that Full Feed clams were significantly heavier than all other treatments, No Feed clams were significantly lighter, and One Week and One Month regimes did not differ (*p* > 0.05).

Together, length and weight responses indicate that feed availability, rather than habitat structure, was the dominant driver of somatic growth in *L. pedicellatus* under the present cultivation conditions.

### Nutritional profile and fatty acid composition of *Lyrodus pedicellatus*

**Fig. 3:**
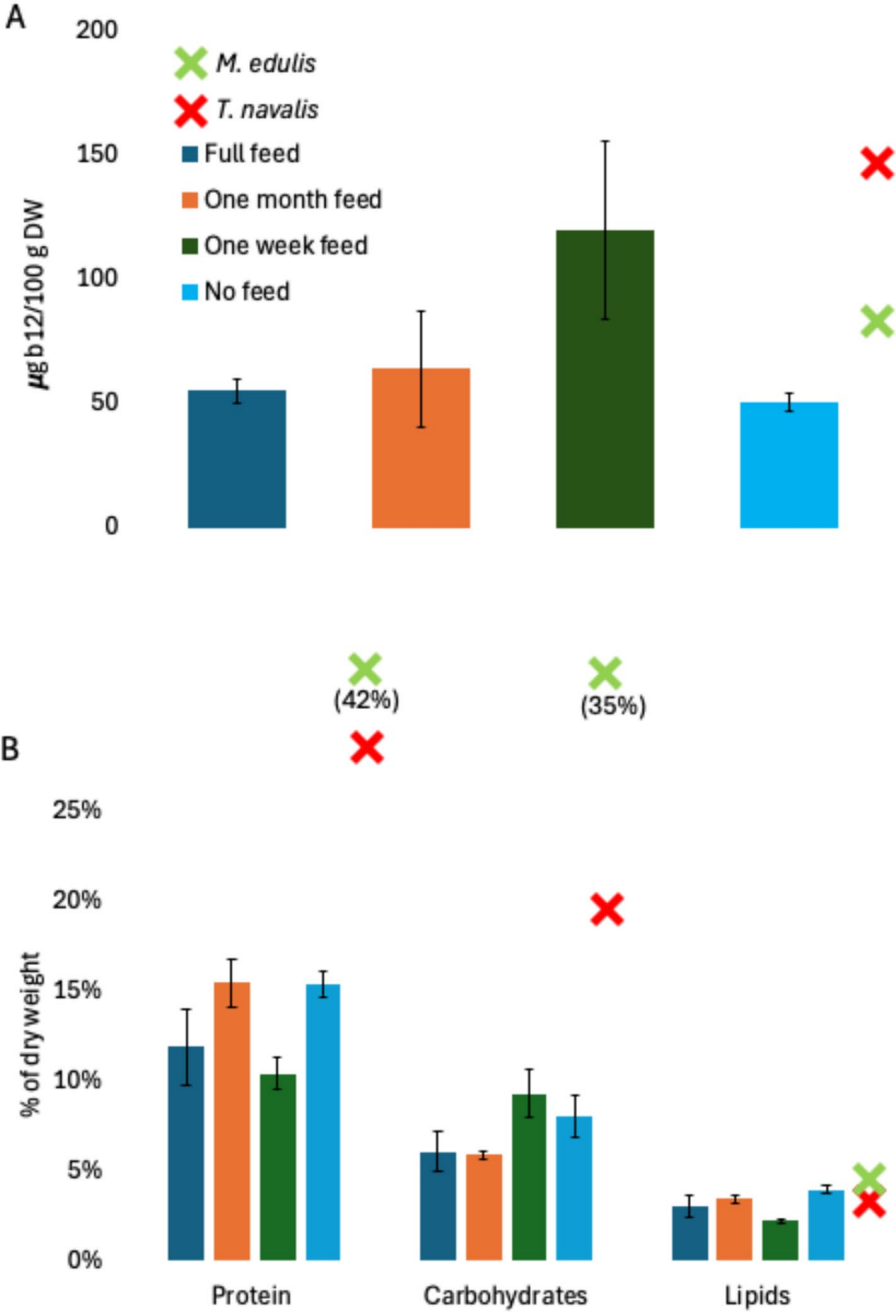
Macronutrient and vitamin B12 profile of** Lyrodus pedicellatus**. Bars show mean protein, carbohydrate, lipid, and B12 content. The error bars represent the standard deviation of three or two biological replicates and three technical replicates. Green x (*Mytilus edulis*) and red × (*Teredo navalis*) are mean data points from Willer et al. 2023. Bars show mean ± SD of biological replicates (*n* = 3 for Full Feed and One Month treatments; *n* = 2 for No Feed and One Week treatments)

**Fig. 4:**
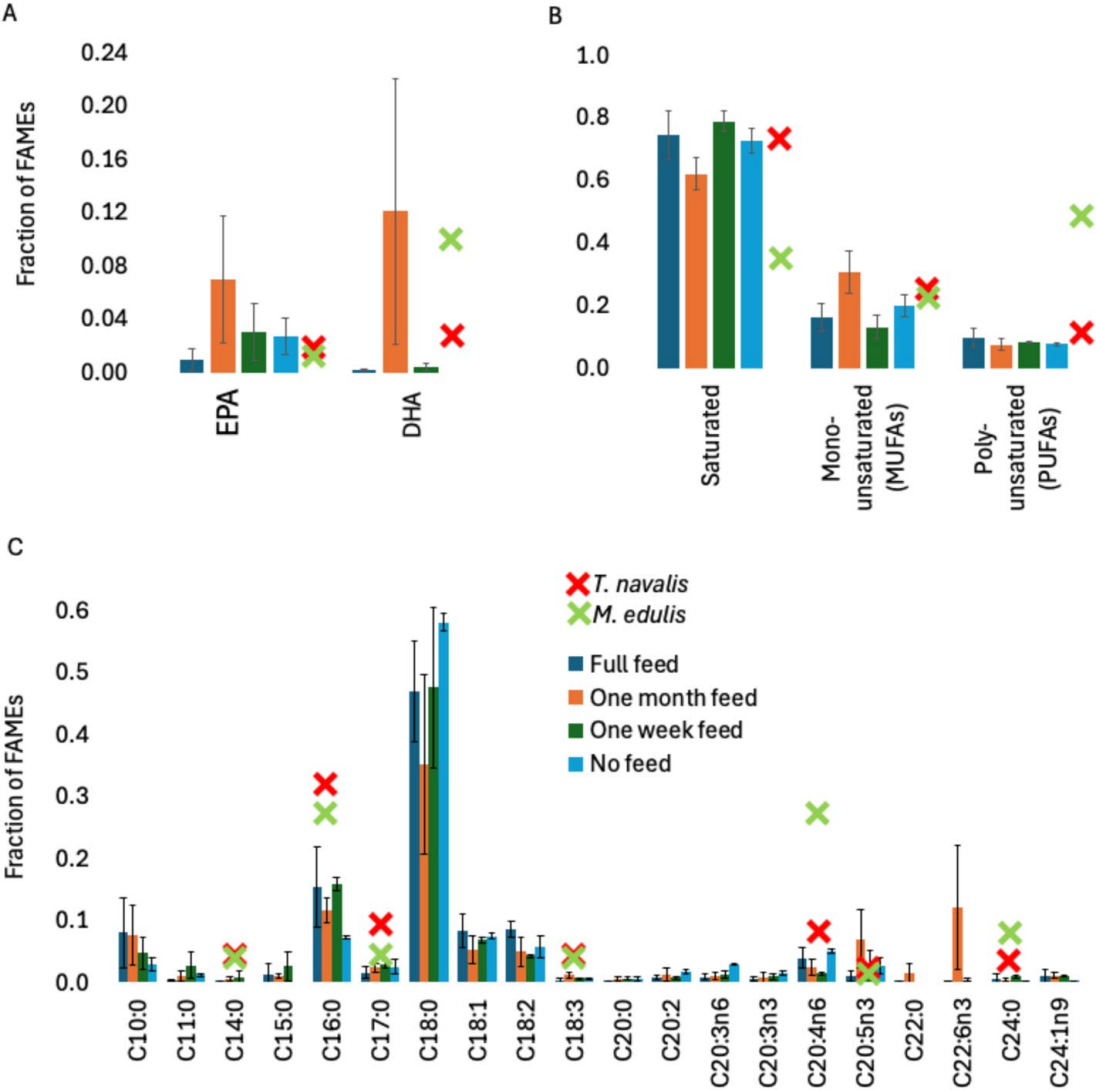
Fatty acid profile of naked clams.** A** EPA (Eicosapentaenoic acid, 20:5(n-3)) and DHA (Cis-4,7,10,13,16,19-docosahexaenoic). **B** Total saturated, mono-unsaturated (MUFAs) and poly-unsaturated (PUFAs). **C** FAMEs composition (expressed as a fraction of total FAMEs) of juvenile naked clams. Values represent mean ± SD of biological replicates (*n* = 3 for Full Feed and One Month treatments; *n* = 2 for No Feed and One Week treatments), based on two or three technical replicates per biological replicate depending on biomass availability. All peaks within the 0.5%RT (retention time) were included in the analysis. Green × (*Mytilus edulis*) and red × (*Teredo navalis*) are mean data points from Willer et al., (2023)

Neither feeding regime nor wood structure influenced the nutritional composition of *L. pedicellatus* (Fig. 3). Vitamin B12 concentration did not differ significantly among feeding treatments (F(3,6) = 3.74, *p* = 0.0794), with a mean value of 69.59 ± 4.0 µg g⁻^1^ dry weight across all samples (Fig. 3A). This level is consistent with previously reported high B12 concentrations in *T. navalis* and blue mussels. Similarly, no significant differences among feeding regimes were detected for macronutrient composition (Fig. 3B). Across treatments, protein content averaged 13.34 ± 0.85% dry weight (F(3,6) = 4.24, *p* = 0.0628); carbohydrate content 7.06 ± 0.57% (F(3,6) = 3.76, *p* = 0.0788); and lipid content 3.21 ± 0.24% (F(3,6) = 4.32, *p* = 0.0605). Fatty acid class composition was likewise invariant across feeding regimes (Fig. 4A, B, C). Saturated fatty acids accounted for 0.71 ± 0.03 of total FAMEs, monounsaturated fatty acids for 0.21 ± 0.03, and polyunsaturated fatty acids for 0.08 ± 0.01, with no significant treatment effects (Fig. 4B). Profiles were characterised by high relative abundances of palmitic acid (C16:0), stearic acid (C18:0), and particularly oleic acid (C18:1) (Fig. 4C), alongside detectable EPA and DHA (Fig. 4A). Overall fatty acid composition closely resembled that reported previously for *T. navalis*.

## Discussion

### Extending the modular system from concept to full life-cycle rearing

Our previous work established that a modular, static-tank system could maintain adult *Teredo navalis* while utilising microencapsulated feeds to fortify its nutritional profile (Willer et al. 2023). In this study, we advance this framework by demonstrating that *Lyrodus pedicellatus* larvae can successfully settle, metamorphose, and grow to maturity within the same system without the requirement for flow-through seawater. This represents a substantial progression from simple adult maintenance to full life-cycle rearing with quantified growth performance. Consequently, these results confirm that a low-infrastructure, static configuration is sufficient to support the post-settlement development of naked clams in both laboratory and remote settings.

Conventional bivalve hatcheries typically rely on flow-through seawater, aeration, extensive water treatment, and on-site algal production (Helm and Bourne 2004; Handå et al. 2013). In contrast, our modular system uses static artificial seawater, wood substrates, and microencapsulated feeds in small-footprint tanks. This simplicity is attractive for deployment in regions with limited infrastructure, aligning with proposals that naked clam aquaculture could provide low-input, locally adaptable blue food (Willer and Aldridge 2020; Willer et al. 2023; Poon et al. 2025).

### Quantification of growth responses to microencapsulated feeds

While Willer et al. (2023) showed that Biobullets are assimilated by *T. navalis* and can fortify tissue nutrient composition, our results provide the first, to our knowledge, quantitative evidence that microencapsulated feeds can drive growth in a primarily xylophagous bivalve. Full Feed clams were significantly longer and heavier than those under all other regimes, whereas No Feed clams were shortest and lightest. One Week and One Month regimes, which provided only limited periods of supplementation, produced intermediate sizes that did not differ significantly.

These findings demonstrate that continuous Biobullet supplementation can substantially enhance growth even when wood is the primary energy source. The likely mechanism is supplementation of easily assimilable lipids, nitrogen, and micronutrients on top of wood-derived carbon and symbiont-mediated nitrogen fixation (Altamia et al. 2021; Willer et al. 2023; Goodell et al. 2024). The fact that growth responses were detectable in static tanks with relatively low water exchange suggests that microencapsulated feeds can be effectively delivered to infaunal bivalves, with potential relevance for other burrowing or structure-associated taxa.

In contrast, wood structure (sheet versus square dowel) had no detectable effect on growth within the present design. This may reflect similar effective surface area and wood quality between the two formats or the dominant role of feed intensity under these controlled conditions. However, field and experimental studies have shown strong effects of wood species, density, chemistry, and grain orientation on shipworm settlement and boring rates (Kohlmeyer et al. 1995; Paalvast and van der Velde 2011; Tagliapietra et al. 2019). Optimising wood type and geometry remains an important avenue for improving growth and harvest efficiency.

### Nutritional generality and new functional fatty acids

The nutritional analyses show that *L. pedicellatus* has a macronutrient and vitamin B12 profile consistent with that reported for *T. navalis*, supporting the view that high nutritional value is shared across teredinid genera rather than confined to a single species (Willer et al. 2023; Poon et al. 2025). Although absolute protein and lipid percentages were somewhat lower than published values for *T. navalis* grown under different conditions (Willer et al. 2023), *L. pedicellatus* still provides meaningful amounts of protein, energy, and micronutrients. Vitamin B12 concentrations around 70 µg g⁻^1^ dry weight are high compared with many conventional seafoods (Lander et al. 2012; Willer et al. 2023), reinforcing the potential of naked clams to address micronutrient deficiencies (Willer and Aldridge 2019; Passarelli et al. 2024).

Fatty acid class composition was broadly similar to *T. navalis*, with high proportions of palmitic and stearic acids, substantial oleic acid, and measurable EPA and DHA (Willer et al. 2023). The detection of nervonic acid (C24:1n9) in *L. pedicellatus* is particularly noteworthy, as this long-chain monounsaturated fatty acid is a vital component of mammalian myelin and is increasingly recognised for its neuroprotective properties such as memory and cognitive function (Li et al. 2019). Together, these traits point to *L. pedicellatus* as a functional food candidate, capable of contributing high-quality protein, B12, and beneficial fatty acids to human diets (Willer et al. 2023; Poon et al. 2025).

That Biobullet supplementation modulated growth but did not significantly alter proximate or fatty acid composition under the tested conditions suggests that nutritional density is relatively robust to moderate variation in feed regime. This contrasts with *T. navalis*, where diet manipulation produced stronger fortification signals (Willer et al. 2023), and highlights the need for species-specific optimisation of microencapsulated feeds. These nutritional findings should, however, be interpreted cautiously given the relatively small number of biological replicates in some treatments (*n* = 2 for No Feed and One Week; *n* = 3 for Full Feed and One Month). While the consistency of the overall patterns is encouraging, larger-scale studies will be needed to confirm treatment effects on biochemical composition and fatty acid profiles with greater statistical power.

### Leveraging growth rates to map future development

Despite significant relative differences among treatments, absolute growth rates in the modular system were lower than literature values reported for *L. pedicellatus* and related species in other contexts (Gallager et al. 1981; Poon et al. 2025). These comparisons should be interpreted cautiously, because historical records attributed to *L. pedicellatus* predate recognition of the cryptic species complex and may include observations on other pedicellatus-like *Lyrodus* taxa. From a commercial perspective, however, further work is required to optimise growth rate of this species to support economically viable production cycles.

Several factors likely contributed to lower-than-expected growth rates. Static 2-L tanks with weekly half-water changes may have limited oxygen availability and waste removal at higher biomasses. Wood quality and conditioning—including pine species, density, prior microbial colonisation, and leached compounds—are known to influence shipworm settlement and boring (Nair and Saraswathy 1971; Kohlmeyer et al. 1995; Tagliapietra et al. 2019), and our substrate may not have been optimal. Temperature and salinity were maintained at 20 °C and typical seawater levels, but may not coincide with the optimum for *L. pedicellatus* growth observed in other studies (Borges et al. 2014; Gallager et al. 1981). Feed dose (5 mg L⁻^1^ day⁻^1^) was also intentionally conservative to minimise pseudofaeces; higher or life-stage-specific doses may yield faster growth.

Importantly, these factors were unapparent in previous work focused on adult survival and composition of pre-grown *T. navalis* (Willer et al. 2023), signalling that *L. pedicellatus* may not be the optimal first commercial species unless its expected growth performance can be restored under controlled conditions. Other teredinids, such as *Bankia gouldi*, *B. setacea*, or *Nausitora hedleyi*, which exhibit very rapid growth and large sizes in field studies (Poon et al. 2025), may offer more favourable production potential, assuming similarly strong nutritional profiles.

Notwithstanding the modest growth rates observed herein, the tractability of *L. pedicellatus* within a relatively simple system—characterised by a continuous breeding cycle and rapid generation times—positions the species as a vital model organism for naked clam aquaculture. Case in point, we successfully demonstrated the efficacy of this model system through trials on different growth substrates, such as square dowel panels, alongside diverse dietary regimes. The technical efficiency and systemic adaptability of this species facilitate rapid aquaculture optimisation that may prove generalisable across the Teredinidae family—for example, rapid testing of different settlement and growth substrates, feed regimes, or aquaculture conditions. Furthermore, *L. pedicellatus* offers an accelerated pathway towards genetic refinement; by identifying and propagating high-performance lines tailored for modular systems, the natural growth advantages of this species may be amplified to meet future industrial demands.

### Roadmap for next steps

Our results suggest several priorities for future naked clam aquaculture research. Feed formulations and delivery regimes should be optimised for teredinids, potentially tailoring particle size, composition and dosing to different life stages and species (Willer and Aldridge 2017, 2019; Willer et al. 2020, 2023). Modular system design should be refined to manage water quality, oxygenation, stocking density, and wood geometry more effectively. In the present study, larval settlement was constrained by the structural design of the culture panels, with plastic barrier plates and sheaths restricting settlement to defined exposed wooden surfaces. However, settlement density within these surfaces was not actively standardised or manipulated. Future work should therefore investigate how initial larval settlement density and subsequent culture stocking density interact with wood geometry, panel design, and feeding regimes to optimise growth performance and production efficiency. A broader portfolio of candidate species should be evaluated for growth and nutritional traits (Poon et al. 2025), allowing the development of a robust shortlist tailored to different regions and resource contexts. Finally, naked clam aquaculture is uniquely positioned to drive circular bioeconomy models by transforming low-value lignocellulosic waste into high-value protein (Willer et al. 2023; Shipway et al. 2024; Poon et al. 2025). Integrating production with forestry byproducts, decommissioned timber products, or agricultural residues—materials often destined for landfill or incineration—could significantly lower the environmental footprint of protein production while providing a stable, low-cost feedstock that does not compete with terrestrial food crops. Ultimately, the maturation of this sector from pilot-scale experiments to industrial reality offers a transformative pathway to address the persistent challenges of global malnutrition and environmental degradation within a circular blue economy.

## Conclusion

Herein, we demonstrate that *Lyrodus pedicellatus* can be reared from larval settlement to maturity within a simple, modular static-tank system, marking a significant transition from earlier work on adult maintenance to comprehensive life-cycle cultivation. Our findings provide the first quantitative evidence that Biobullet algal feeds significantly increase both length and wet weight in these bivalves, confirming that supplemental algal feeds can substantially enhance growth beyond that provided by a primarily wood diet. The nutritional density of *L. pedicellatus* proved robust to variations in feed regime, offering a profile rich in protein, vitamin B12, and neuroprotective nervonic acid (C24:1n9). Notably, the consistency of these findings with existing data for other teredinid species suggests that exceptional nutritional density may be a general characteristic of the Teredinidae family which would be consistent with widespread anecdotal reports on their health benefits.

The biological tractability and year-round reproductive capacity of this species position it as a vital model organism for the sector. Case in point, the efficacy of our modular system for testing different substrates and dietary inputs provides a clear blueprint for iterative optimisation that may be generalisable across the family. For example, our system provides a practical platform for future optimisation of feed formulations, wood substrates, stocking densities, and water management, as well as for the comparative screening of additional candidate species. Strategic priorities include the development of high-performance genetic lines – specifically selecting for *L. pedicellatus* individuals that exhibit superior wood-boring rates and feed conversion efficiency within modular environments—alongside the refinement of water quality management. By coupling these technical advancements with industrial symbiosis, specifically the integration of forestry and agricultural waste streams, naked clam aquaculture offers a transformative, low-carbon pathway to support human nutritional requirements. This work therefore establishes a robust experimental foundation for future optimisation and scaling of naked clam aquaculture systems capable of converting lignocellulosic substrates into nutritionally valuable seafood.

## Data Availability

All data is available in the manuscript files.
